# Announcement: Vth Conference of the International Society for Trace Element Research in Humans (ISTERH) *“ New Aspects of Trace Element Research”*

**DOI:** 10.1155/MBD.1998.115

**Published:** 1998


					Vth CONFERENCE
OF THE INTERNATIONAL SOCIETY FOR TRACE
ELEMENT RESEARCH IN HUMANS (ISTERH)
"New Aspects of Trace gIement Research "
Trace Element- Institute for UNESCO, Lyon, France
26- 30th September 1998
Nutrition bioavailability, problems in developing countries, dietary
recommendations, epidemiological aspects.
Pathological and therapeutic aspects cancer, degenerative disorders, bone
disease, diabetes.
Genetics copper disorders, hemochromatosis, metals and gene expression.
Reactive oxygen species genotoxicity, apoptosis, aging, anti-oxidants.
Pediatric aspects:growth, immunity.
Geo-environment cardiomyopathy and selenium, fluorosis.
, Analytical aspects: quality assurance, speciation.
Official languages" French and English (simultaneous translation)
Accepted abstracts will be published in" J. Trace Elements in Experimental
Medicine.
For further information, contact"
Trace Element- Institute for UNESCO
1 place de I'Ecole, BP 7021,
F-69342 LYON Cedex 07, France.
Tel +33 4 72 80 82 90 Fax: +33 4 78 58 86 71
E-mail" 101711.2322 @compuserve.com
trace,elem.for,unesco @wanadoo.fr
115

				

